# Seeing the Future of Lung Cancer Vaccination

**DOI:** 10.3390/ijms252111521

**Published:** 2024-10-26

**Authors:** Kostas A. Papavassiliou, Amalia A. Sofianidi, Vassiliki A. Gogou, Athanasios G. Papavassiliou

**Affiliations:** 1 First University Department of Respiratory Medicine, ‘Sotiria’ Chest Hospital, Medical School, National and Kapodistrian University of Athens, 11527 Athens, Greece; konpapav@med.uoa.gr (K.A.P.); gogvanessa@gmail.com (V.A.G.); 2 Department of Biological Chemistry, Medical School, National and Kapodistrian University of Athens, 11527 Athens, Greece; amsof.00@gmail.com

It has been nearly fifteen years since the Food and Drug Administration (FDA) approved the first therapeutic cancer vaccine for solid tumors, namely Sipuleucel-T (Provenge**^®^**), marking a significant milestone in the treatment of metastatic castration-resistant prostate cancer [1]. Since then, only one other therapeutic cancer vaccine has received FDA approval for solid tumors—Talimogene Laherparepvec (T-VEC or Imlygic)—which is a groundbreaking oncolytic virus (genetically modified herpes simplex virus (HSV)) therapy designed to treat advanced melanoma [2]. However, despite numerous clinical trials investigating the potential of cancer vaccines for the treatment of lung cancer [3], until recently, the results have been largely disappointing. This challenging and lethal disease has proven difficult to target effectively with vaccine-based therapies, which is mainly attributed to its highly immunosuppressive tumor microenvironment (TME) [4]. Developing vaccines to combat lung cancer involves tipping the balance between immune activation and immune suppression toward enhanced immune activation, highlighting the need for continued innovation and research in this field.

One of the most exciting developments in lung cancer treatment is the creation of personalized vaccines that target neoantigens—unique, mutated proteins found exclusively on a patient’s tumor cells [5]. Unlike tumor-associated antigens (TAAs), which can be present in both tumor and normal tissues, neoantigens are tumor-specific antigens (TSAs) that are solely expressed in cancerous cells. This distinction allows for a more precise targeting of the immune response, potentially leading to more effective and tailored therapies for individual patients [6]. By employing whole-exome sequencing techniques, researchers can identify individual-specific neoantigens that serve as promising candidates for lung cancer vaccination [7,8]. The integration of artificial intelligence (AI) in this process holds significant potential as it can aid in pinpointing the neoantigens that are likely to be highly immunogenic, enhancing the effectiveness of personalized cancer vaccines.

Since the success of COVID-19 mRNA vaccines, a promising new avenue has opened, i.e., the use of mRNA technology to treat lung cancer. BioNTech has taken a pioneering step by developing BNT116, the world’s first mRNA vaccine for lung cancer. This inventive vaccine targets non-small-cell lung cancer (NSCLC), the most prevalent form of the disease. BNT116 is an intravenously administered uridine RNA-based lipoplex cancer vaccine comprising six mRNAs (MAGE A3, CLDN6, KK-LC-1, PRAME, MAGE A4, and MAGE C1), each encoding a TAA that is frequently expressed in NSCLC, aiming to stimulate a precise and robust immune response against tumor cells. There is great optimism around combining BNT116 with existing immunotherapies, potentially fostering the immune system’s ability to target and destroy cancer cells quickly and more effectively [9]. Vaccines can prime the immune system, and immune checkpoint inhibitors (ICIs), for example, can sustain the immune response, potentially leading to better outcomes.

Peptide vaccines constitute the majority of lung cancer vaccines currently being tested in clinical trials. Nevertheless, the results from phase III trials have been largely dispiriting [3]. To address this, efforts are being focused on incorporating different adjuvants to enhance the immunogenicity of peptide-based therapeutic vaccines, with the intention of boosting their effectiveness in treating lung cancer [10]. Ongoing preclinical trials also aim to reshape the future of clinical approaches to lung cancer vaccination. These studies have shown, for instance, that a single-peptide vaccine targeting anaplastic lymphoma kinase (ALK), a receptor tyrosine kinase responsible for the regulation of cellular proliferation and differentiation, can restore the function of impaired CD8+ T cells (which play a crucial role in immune surveillance and defense against cancer). When combined with tyrosine kinase inhibitors (TKIs), this strategy has effectively eliminated ALK-positive (i.e., the rearrangement of the *echinoderm microtubule-associated protein-like 4* (*EML4*) gene and the *ALK* gene generating fusion oncogene *EML4-ALK*) NSCLC [11]. At present, this promising vaccine approach is being evaluated in an ongoing phase I/II clinical trial for advanced ALK-mutated NSCLC (NCT05950139), underlining its potential to transform the future of lung cancer treatment.

Beyond the therapeutic promise of lung cancer vaccines, there is hope for the development of preventive vaccines in the future, akin to how vaccination has successfully prevented human papillomavirus (HPV)-induced cervical cancer [12]. Although lung cancer is not caused by a virus like cervical cancer, researchers are exploring strategies to create vaccines that target the underlying mechanisms leading to the disease, such as mutations triggered by smoking-related carcinogens [13]. These efforts could pave the way for reducing lung cancer risk before it even develops.

In conclusion, the future of lung cancer vaccines is poised at a thrilling juncture, driven by advances in genomics, mRNA technology, and AI. Therapeutic vaccines are becoming more personalized, targeting unique neoantigens to deliver precise, efficacious treatments, while the prospect of a preventive vaccine gives hope for a future where lung cancer can be mitigated before it starts. Although challenges remain, the field is rapidly evolving, and each step forward brings us closer to transforming lung cancer care, offering renewed hope to patients and their families worldwide.

## References

[1] Kantoff P.W., Higano C.S., Shore N.D., Berger E.R., Small E.J., Penson D.F., Redfern C.H., Ferrari A.C., Dreicer R., Sims R.B. (2010). Sipuleucel-T Immunotherapy for Castration-Resistant Prostate Cancer. N. Engl. J. Med..

[2] Andtbacka R.H.I., Kaufman H.L., Collichio F., Amatruda T., Senzer N., Chesney J., Delman K.A., Spitler L.E., Puzanov I., Agarwala S.S. (2015). Talimogene Laherparepvec Improves Durable Response Rate in Patients With Advanced Melanoma. J. Clin. Oncol..

[3] Sanaei M.-J., Pourbagheri-Sigaroodi A., Rezvani A., Zaboli E., Salari S., Masjedi M.R., Bashash D. (2024). Lung Cancer Vaccination from Concept to Reality: A Critical Review of Clinical Trials and Latest Advances. Life Sci..

[4] Genova C., Dellepiane C., Carrega P., Sommariva S., Ferlazzo G., Pronzato P., Gangemi R., Filaci G., Coco S., Croce M. (2022). Therapeutic Implications of Tumor Microenvironment in Lung Cancer: Focus on Immune Checkpoint Blockade. Front. Immunol..

[5] Minati R., Perreault C., Thibault P. (2020). A Roadmap Toward the Definition of Actionable Tumor-Specific Antigens. Front. Immunol..

[6] Xie N., Shen G., Gao W., Huang Z., Huang C., Fu L. (2023). Neoantigens: Promising Targets for Cancer Therapy. Signal Transduct. Target. Ther..

[7] Karasaki T., Nagayama K., Kawashima M., Hiyama N., Murayama T., Kuwano H., Nitadori J., Anraku M., Sato M., Miyai M. (2016). Identification of Individual Cancer-Specific Somatic Mutations for Neoantigen-Based Immunotherapy of Lung Cancer. J. Thorac. Oncol..

[8] Su S., Chen F., Xu M., Liu B., Wang L. (2023). Recent Advances in Neoantigen Vaccines for Treating Non-small Cell Lung Cancer. Thorac. Cancer.

[9] Deme D., Öven B., Göker E., Lang I., Brück P., Wenger M., Munshi N., Schell T., Ünsal-Kaçmaz K., Markman J. (2023). 597 Preliminary Results from LuCa-MERIT-1, a First-in-Human Phase I Trial Evaluating the Fixed Antigen RNA Vaccine BNT116 in Patients with Advanced Non-Small Cell Lung Cancer. J. ImmunoTherapy Cancer.

[10] Marriott M., Post B., Chablani L. (2023). A Comparison of Cancer Vaccine Adjuvants in Clinical Trials. Cancer Treat. Res. Commun..

[11] Mota I., Patrucco E., Mastini C., Mahadevan N.R., Thai T.C., Bergaggio E., Cheong T.-C., Leonardi G., Karaca-Atabay E., Campisi M. (2023). ALK Peptide Vaccination Restores the Immunogenicity of ALK-Rearranged Non-Small Cell Lung Cancer. Nat. Cancer.

[12] Falcaro M., Soldan K., Ndlela B., Sasieni P. (2024). Effect of the HPV Vaccination Programme on Incidence of Cervical Cancer and Grade 3 Cervical Intraepithelial Neoplasia by Socioeconomic Deprivation in England: Population Based Observational Study. BMJ.

[13] Centers for Disease Control and Prevention (US), National Center for Chronic Disease Prevention and Health Promotion (US), Office on Smoking and Health (US) (2010). How Tobacco Smoke Causes Disease: The Biology and Behavioral Basis for Smoking-Attributable Disease: A Report of the Surgeon General.

